# Benign peripheral nerve sheath tumor of digit versus major-nerve: Comparison of MRI findings

**DOI:** 10.1371/journal.pone.0230816

**Published:** 2020-03-26

**Authors:** Seul Ki Lee, Jee-Young Kim, Hyang Sook Jeong

**Affiliations:** 1 Department of Radiology, St. Vincent’s Hospital, College of Medicine, The Catholic University of Korea, Seoul, Republic of Korea; 2 Department of Hospital Pathology, St. Vincent’s Hospital, College of Medicine, The Catholic University of Korea, Seoul, Republic of Korea; McLean Hospital, UNITED STATES

## Abstract

**Objective:**

To compare the symptoms and magnetic resonance imaging (MRI) findings between digital peripheral nerve sheath tumor (PNST) and major-nerve PNST.

**Methods:**

A total 36 cases with benign PNSTs (16 digital, 20 major-nerve) were enrolled. Chief complaint and Tinel sign were reviewed. Five classic MRI features of PNST, the signal intensity (SI), the enhancement, and the shape of tumor were evaluated on MRI.

**Results:**

Half of each group showed tenderness. Tinel sign was less frequent in digital PNST (12.5%) than major-nerve PNST (95.0%, *P* < 0.001). Split fat sign, entering and exiting nerve, target sign, and thin hyperintense rim were only observed in major-nerve PNST (*P* = <0.001, <0.001, 0.492, and 0.002, respectively). Fascicular sign was found in digital PNSTs (31.3%), but more frequent in major-nerve PNST (*P* < 0.001). In digital PNSTs, mild hyperintense SIs (56.2%) on T1-weighted images (T1-WI) was noted, but none in major-nerve PNST (*P* < 0.001). Both groups showed hyperintense SIs on T2-WI (*P* = 0.371). Homogeneity on T2-WI was noted in 43.8% of digital PNSTs, but none in major-nerve PNSTs (*P* = 0.004). Both groups showed heterogeneous enhancement (*P* = 0.066), but four (25%) digital PNSTs showed homogeneous enhancement. Lobulated shape was noted in 50% of digital PNSTs but none of major-nerve PNSTs (*P* = 0.001). Digital nerve was involved at 81.3% of digital PNSTs. Three foot cases showed unusual manifestations: bone destruction, skin thickening, and subungual location.

**Conclusion:**

In digital PNSTs, Tinel sign is not commonly found and classic MRI findings is insufficient. In addition, some digital PNSTs show different SI and enhancement from major-nerve PNSTs. However, digital soft tissue tumor involving digital neurovascular bundle and especially representing a fascicular sign should be considered the possibility of a digital PNST.

## Introduction

Various soft tissue masses may be encountered in the digit, and the majority are benign [1, 2]. The most common soft tissue masses in the digit are ganglions followed by tenosynovial giant cell tumor (GCT) [3]. Most soft tissue masses in the digit occur as a small nodule which may or may not present clinical symptoms. Pain or tenderness are not characteristic of any differential diagnosis [4]. Although uncommon, a peripheral nerve sheath tumor (PNST) of the hand or foot can occur, but PNST in the digit is extremely rare [1, 5]. Therefore, PNST is not always included in the differential diagnosis for a soft tissue mass of the digit.

PNST presents characteristic clinical manifestations and magnetic resonance imaging (MRI) findings, and they are usually found in a major peripheral nerve [6, 7]. In the hand and wrist, schwannomas arise from deeper larger nerves, often the flexor surfaces, whereas neurofibromas tend to involve smaller cutaneous nerves [8]. PNSTs predominantly occur in patients 20–40 years of age with no sex predilection. In some reports, 10.3–11.5% of PNSTs were located in the foot or ankle [9]. Schwannomas represent the most common histologic type [10]. If a major nerve is involved, it may have associated neurologic findings, such as Tinel sign [11]. However, a digital PNST presents as a slow-growing, painless, movable mass [8]. Classic MRI findings, useful for the evaluation of PNSTs, include the split-fat sign, target sign, fascicular pattern, entering and exiting nerve, and a thin hyperintense rim [7, 12–15]. However, only a few cases of digital PNSTs have been reported, so there is less information on whether digital PNSTs reveal characteristic MRI findings or the prevalence of these findings.

The purpose of this study was to compare the symptoms and MRI findings between digital PNST and major-nerve PNST. We also described the possible differential diagnoses of digital PNST.

## Material and methods

### Study population

This retrospective study was approved by St. Vincent’s Hospital’s Institutional Review Board, and the requirement for informed consent was waived. Our objective was to compare two groups: digital benign PNST (the study group) and major-nerve benign PNST (the control group). The cases of digital benign PNST were selected consecutively from August 2004 to June 2019 during which 660 MRIs of PNSTs were performed. Among this group of subjects who underwent MRI for PNST, we identified 16 cases of digital benign PNST involving an area from the digital phalangeal level to the web space level where each common digital nerve is divided into proper digital nerves [3, 16]. Subjects from the control group of 20 cases of major-nerve benign PNSTs were selected out of the MRIs performed in our institution with inclusion criteria of benign PNST involving the major nerves of the upper or lower extremities during the same study period. Patients were excluded if they had a mass in the subcutaneous fat layer, within the muscle, or along spinal nerve roots. A total of 36 cases with pathologically-proven benign PNSTs (n = 16 in a digit; n = 20 in a major nerve) were enrolled in this study. The patients consisted of 15 males and 21 females (mean age, 50.5 years; range, 15–78 years). None of patients was associated with neurofibromatosis. Each patient’s clinical presentations of tenderness and Tinel sign were reviewed.

### MRI acquisition

MRI was performed with 1.5-T (Gyroscan Intera or Ingenia, Philips Healthcare, The Netherlands) and 3.0-T (Magnetom Verio, Siemens Healthineers, Germany) scanners. MRI protocols included spin-echo T1-weighted (TR/TE range, 370–693/10-19 in 1.5-T, 623/11 in 3.0-T), spin-echo T2-weighted with and without fat suppression (TR range/TE range, 1648–3280/80–100 in 1.5-T, 4000–6200/63–76 in 3.0-T), and gadolinium enhancement of T1-weighted images with fat suppression. Axial, coronal, and sagittal images were obtained in all patients. Gadolinium-enhanced T1-weighted images were obtained in at least two orthogonal planes.

### Imaging analysis

Two musculoskeletal radiologists (J.Y.K and S.K.L, with 20 years and 4 years of experience in musculoskeletal radiology, respectively) retrospectively reviewed the MRI features to determine the maximum diameter along both longitudinal axis and short axis and the location of each tumor. On MRI evaluation, classic MRI findings which were often highly suggestive of a PNST were evaluated. Reviewers evaluated the ‘split fat sign’ on T1-weighted images, and they evaluated the ‘fascicular sign’, ‘target sign’, ‘entering and exiting nerve’ and ‘thin hyperintense rim’ on T2-weighted images. All decisions were reached by consensus. The ‘split fat sign’ represents a rim of fat that surrounds a mass, particularly in the proximal and distal ends of the mass [13]. The ‘entering and exiting nerve’ represents a hyperintense signal fiber situated along the longitudinal axis of a mass on T2-weighted images [17]. The ‘fascicular sign’ was defined as multiple, small, hypointense foci interspersed within a hyperintense area on T2-weighted images [13]. The ‘target sign’ was classified when the central hypointense focus comprised less than three-quarters of the lesion’s diameter with a peripheral hyperintense rim on T2-weighted images [15]. The ‘thin hyperintense rim’ was defined as a mass having a thin hyperintense rim that comprised less than one-quarter of the lesion’s diameter on T2-weighted images [15]. Additionally, signal intensity (SI) characteristics, enhancement pattern, shape, and location of a mass were assessed. Skeletal muscle was used as the reference tissue for SI on the T1- and T2-weighted images.

### Histological analysis

After imaging, all patients underwent complete excision of their masses. An experienced pathologist examined the excised specimens, and a final diagnosis was established on the basis of histological morphology and immunohistochemistry.

### Statistical analysis

The chi-square test and Mann-Whitney *U* test were used to compare the findings between the two groups. Logistic regression analysis was performed between two groups against MRI findings in terms of clinical baseline as a confounding factor. Interobserver variability was assessed using kappa statistics. A kappa value of less than 0.40 indicated poor agreement; 0.40 to 0.59, moderate agreement; 0.60 to 0.79, good agreement; and 0.80 or greater, excellent agreement. For all statistical comparisons, the significance level was set at *P* < 0.05. Statistical analysis was conducted using software packages (SPSS v. 20.0, Chicago, IL).

## Results

**Fig 1:**
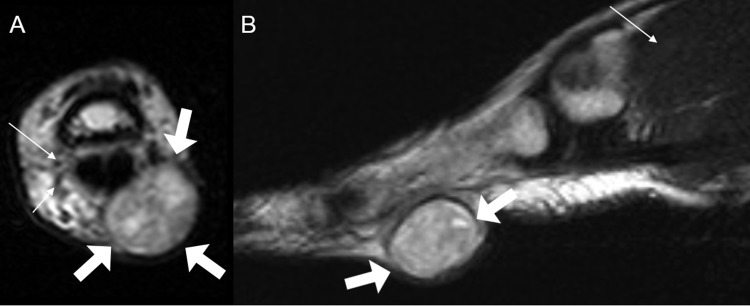
A 31-year-old man with digital schwannoma on the volar side of the 2^nd^ proximal phalanx of the hand. (**A)** On axial T2-weighted image, the mass shows multiple, small, low signal-intensity foci in a background of hyperintensity, representing the fascicular sign (thick arrows). The mass involves the digital neurovascular bundle. A normal palmar digital artery (long thin arrow) and normal palmar digital nerve (short thin arrow) are shown for comparison. (**B)** On sagittal T2-weighted image, the mass shows fascicular sign (thick arrows) in a background of hyperintensity compared to the skeletal muscle (thin arrow).

**Fig 2:**
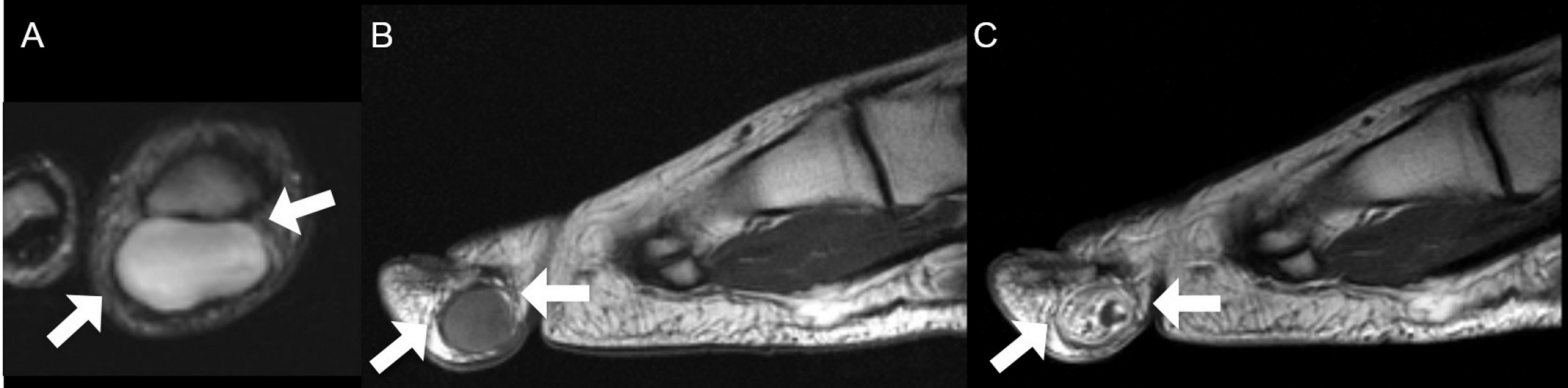
A 15-year-old woman with digital neurofibroma on the plantar side of the 1^st^ distal phalanx of the foot. (**A)** On axial T2-weighted image, the mass shows mild lobulation and bright SI similar to fluid (arrows). (**B)** On sagittal T1-weighted image, the mass shows mild hyperintense SI compared to the skeletal muscle (arrows). (**C)** On sagittal contrast-enhanced T1-weighted image, the mass shows heterogeneously intense enhancement (arrows). This image characteristic correlates well with other reported cases of neurofibroma as iso or slightly hyperintense tissue on T1-weighted images, mildly or markedly hyperintense to skeletal muscle signals on T2-weighted image, and heterogeneous enhancement [18, 19]. ***SI***, signal intensity.

**Fig 3:**
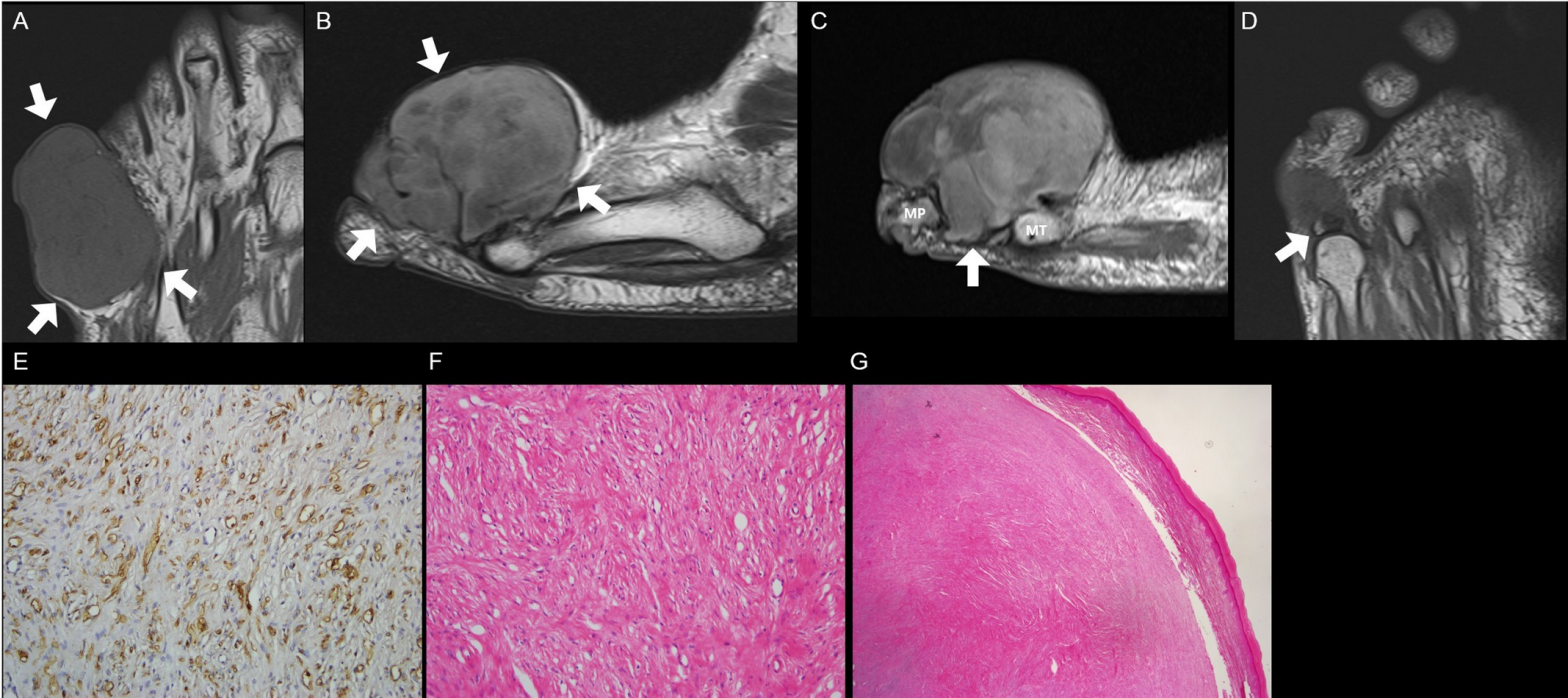
A 59-year old woman with digital ancient schwannoma on the dorsal side of the 5^th^ proximal phalanx of the foot. **(A)** On coronal T1-weighted image, the mass shows isointense SI compared to the skeletal muscle (arrows). (**B)** On sagittal T2-weighted image, the mass reveals lobulation in shape and heterogeneously hyperintense SI compared to skeletal muscle (arrows). (**C)** On sagittal contrast-enhanced T1-weighted image, the mass demonstrates heterogeneous enhancement with bone destruction of proximal phalanx (arrow). (**D)** On coronal T1-weighted image, the proximal phalanx focally remains with preserved normal fatty marrow (arrow). (**E)** Immunohistochemically (S100X200), tumor cells are equivocally positive for S-100 protein. (**F)** Benign neurogenic tumor with hyalinization, suggestive of schwannoma with ancient change (H&E, X200). (**G)** The expanding mass with well encapsulated fibrous capsule is in close proximity to the adjacent epidermis but not invades (H&E, X40). ***SI***, signal intensity.

The final diagnoses, established on the basis of histopathology, were composed of 16 benign PNSTs of the digit (schwannoma, n = 11 [Fig 1]; neurofibroma, n = 4 [Fig 2]; ancient schwannoma, n = 1 [Fig 3]) and 20 benign PNSTs of a major nerve (schwannoma, n = 19; neurofibroma, n = 1). The detailed anatomical information from the two groups is described in Table 1.

**Table 1:** Summary of patient characteristics and clinical findings.

	Digital PNST (n = 16)	Major-nerve PNST (n = 20)	*P*
Age (years)	45.7 ± 16.5	54.4 ± 11.2	0.072
Gender			
male	7 (43.8%)	8 (40.0%)	0.821
female	9 (56.3%)	12 (60.0%)	
Tenderness	8 (50.0%)	11 (55.0%)	1.000
Tinel sign	2 (12.5%)	19 (95.0%)	< 0.001
Location (n)	Hand, web space, volar (1)	Shoulder, axillary n. (2)	
	Hand, PP, dorsal (1)	Upper arm, radial n. (1)	
	Hand, PP, volar (2)	Upper arm, median n. (1)	
	Hand, MP, dorsal (1)	Elbow, ulnar n. (1)	
	Hand, MP, volar (2)	Elbow, radial n. (2)	
	Foot, PP, dorsal (2)	Forearm, median n. (2)	
	Foot, PP, plantar (3)	Forearm, radial n. (1)	
	Foot, MP, dorsal (1)	Wrist, median n. (2)	
	Foot, DP, dorsal (1)	Hip, sciatic n. (1)	
	Foot, DP, plantar (2)	Thigh, saphenous n. (1)	
		Knee, common peroneal n. (5)	
		Ankle, posterior tibial n. (1)	

Data are means ± standard deviations.

DP, distal phalanx; MP, middle phalanx; n., nerve; PP, proximal phalanx.

### Comparison of patient characteristics and clinical manifestations between the two groups

The mean age of patients with digital PNST was 45.7 years and that of patients with major-nerve PNST was 54.4 years (*P* = 0.072). There was no significant difference in the gender of the subjects (*P* = 0.821). There was also no significant difference in the presence of tenderness between the groups (digital PNSTs, 50.0%; major-nerve PNSTs, 55.0%, *P* = 1.000). Tinel sign was more frequent in major-nerve PNSTs (95.0%) and only two cases of digital PNSTs (12.5%) with a significant difference between the two groups (*P* < 0.001). Table 1 demonstrates a summary of the patient characteristics and clinical manifestations of the two groups.

### Comparison of MRI findings between the two groups

The mean maximum diameter of the digital PNST and major-nerve PNST was 10.7 mm and 22.3 mm in the longitudinal axis with significant difference (*P* < 0.001) and 16.2 mm and 17.2 mm in the short axis with no significant difference (*P* = 0.613). The split fat sign was not detected in any digital PNST but was detected in all major-nerve PNST, with statistical difference (*P* < 0.001). No entering and exiting nerves were observed in any digital PNST, while they were observed in 19 (95.0%) major-nerve PNST. This difference was also statistically significant (*P* < 0.001). The fascicular sign was seen in 5 cases (31.3%) of the digital PNST (Fig 1) and in 18 cases (90.0%) of the major-nerve PNST. This difference was statistically significant (*P* < 0.001). Age-adjusted logistic regression analysis showed that the comparison of fascicular sign between two groups was not confounded by age (S1 Table). The target sign was observed in 2 (10.0%) major-nerve PNST but not in digital PNST without statistical difference (*P* = 0.492). The thin hyperintense rim on T2-weighted images was demonstrated in 9 (45.0%) major-nerve PNST but not in digital PNST. This difference was statistically significant (*P* = 0.002). The interobserver agreement for the MRI findings was excellent in split fat sign, entering and exiting nerves, target sign, thin hyperintense rim (kappa = 0.889–1.000), and good in fascicular sign (kappa = 0.769). The MRI features of the two groups are described in Table 2.

**Table 2:** Comparison of MRI findings between digital PNST and major-nerve PNST.

	Digital PNST (n = 16)	Major-nerve PNST (n = 20)	*P*
Maximum diameter (mm)			
longitudinal axis	10.7 ± 3.7	22.3 ± 7.4	< 0.001
short axis	16.2 ± 6.0	17.2 ± 5.2	0.613
Split fat sign	0 (0.0%)	20 (100.0%)	< 0.001
Entering and exiting nerve	0 (0.0%)	19 (95.0%)	< 0.001
Fascicular sign	5 (31.3%)	18 (90.0%)	< 0.001
Target sign	0 (0.0%)	2 (10.0%)	0.492
Thin hyperintense rim	0 (0.0%)	9 (45.0%)	0.002

Data are means ± standard deviations.

We also compared MRI findings between only schwannomas of digital and major-nerve with exception of neurofibroma (S2 Table).

**Fig 4:**
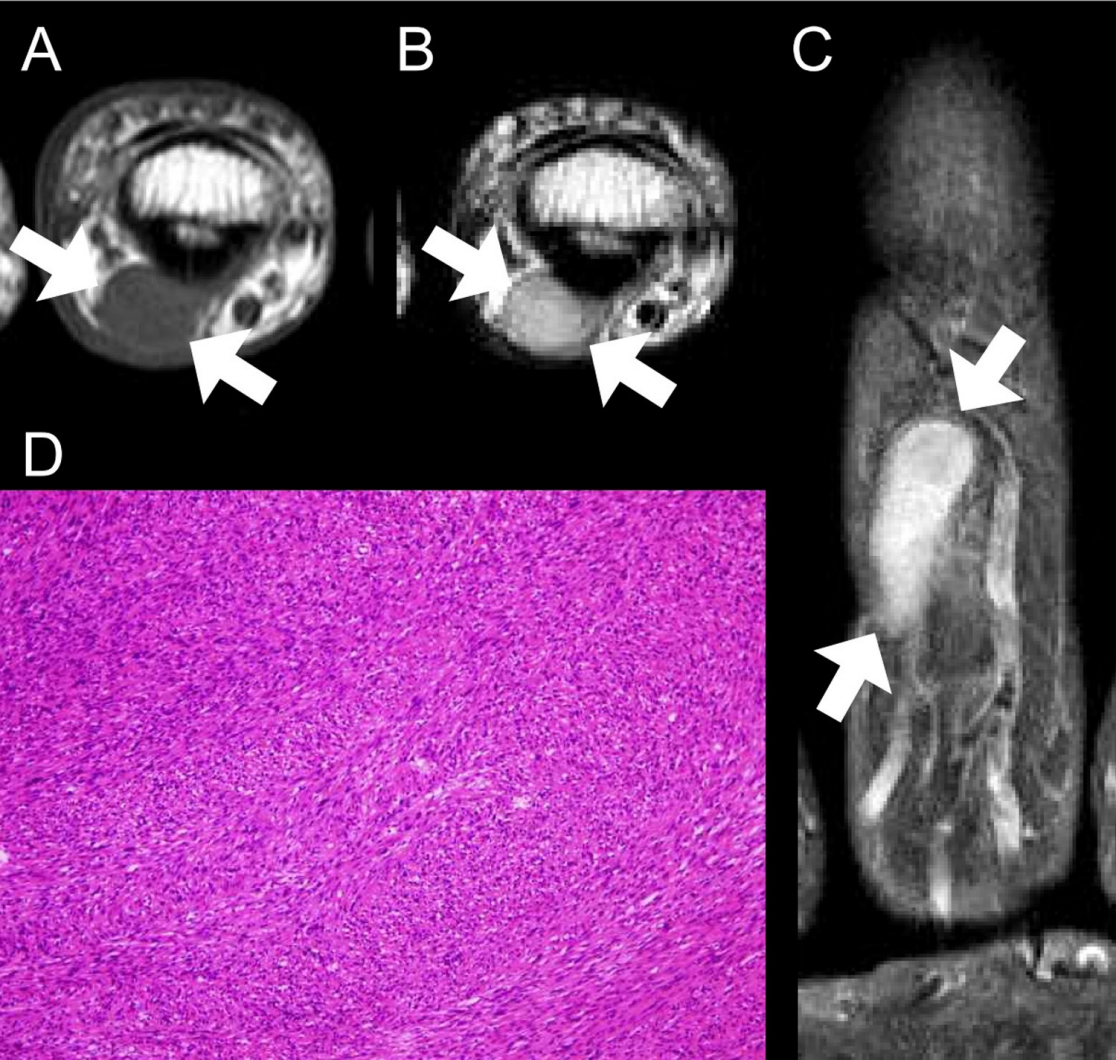
A 56-year old man with digital schwannoma on the volar side of the 3^rd^ middle phalanx of the hand. **(A)** On axial T1-weighted image, the mass shows isointense SI (arrows). **(B)** On axial T2-weighted image, the mass reveals homogeneously hyperintense SI (arrows). **(C)** On coronal contrast-enhanced T1-weighted image, the mass also demonstrates homogeneous enhancement (arrows). **(D)** Tumor contains compact hypercellular components (Antoni A) without typical change of schwannoma (hyaline or cystic change, large size vessel within the tumor) arising from major-nerve (H&E, × 100). ***SI***, signal intensity.

On precontrast T1-weighted images, mildly hyperintense SI compared to the skeletal muscle was noted in nine (56.2%) digital PNSTs (Figs 2 and 6) but none (0.0%) in major-nerve PNSTs with significant difference (*P* < 0.001). On T2-weighted images, hyperintense SI compared to the skeletal muscle was noted in almost of digital PNSTs (15, 93.8%) cases and in all (20, 100.0%) cases of major-nerve PNSTs without significant difference (*P* = 0.444). However, there was one case of digital PNSTs that was noted as bright SI as fluid (Fig 2). Seven (43.8%) digital PNSTs revealed homogeneous SI on T2-weighted images (Figs 2, 4 and 6), however, none in major-nerve PNSTs. This difference was statistically significant (*P* = 0.004). On contrast-enhanced T1-weighted images, heterogeneous enhancement was noted in twelve (75.0%) digital PNSTs and in all (100.0%) major-nerve PNSTs without significant difference (*P* = 0.066). However, there were also four cases (25.0%) of digital PNSTs that were noted as homogeneous enhancement (Figs 4 and 6), which was not revealed in the cases of major-nerve PNSTs.

**Fig 5:**
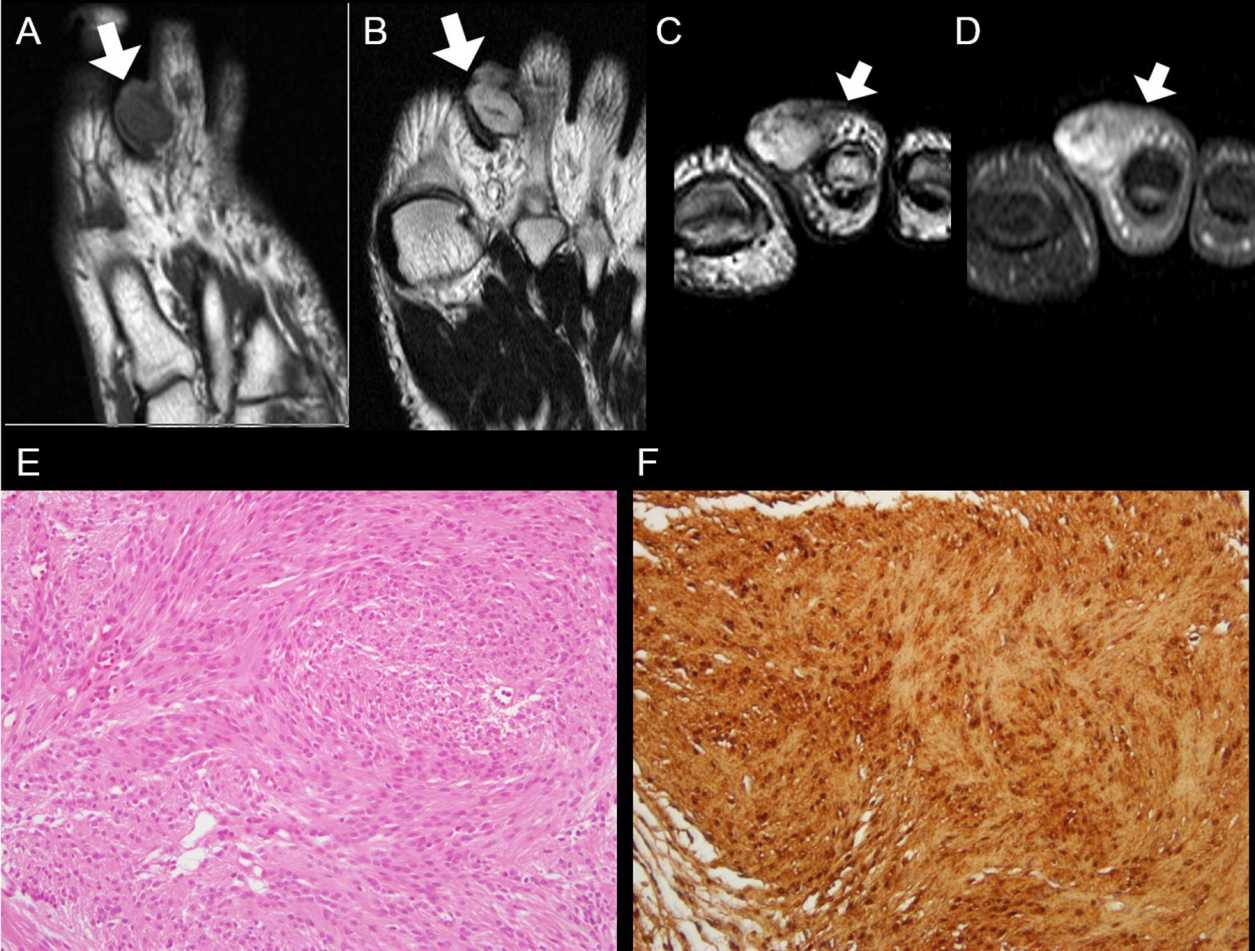
A 54-year old man with digital schwannoma on the dorsomedial side of the 2^nd^ proximal phalanx of the foot. (**A)** On coronal T1-weighted image, the mass demonstrates isointense SI compared to skeletal muscle (arrow). (**B)** On coronal T2-weighted image, the lobulated mass reveals heterogeneously hyperintense SI compared to skeletal muscle (arrow). (**C)** On axial T2-weighted image, the skin adjacent to the mass shows focal thickening (arrow). (**D)** On axial contrast-enhanced T1-weighted image, the mass also reveals heterogeneous enhancement with adjacent skin thickening. (**E)** Schwannoma with vague hypo- and hypercellular alterative lesion with no definite nuclear atypia (H&E, X200). (**F)** Immunohistochemically (S100X200), tumor cells are strongly positive for S-100 protein. On the pathologic report, skin thickening is noted as inflammatory reactive change without skin invasion. ***SI***, signal intensity.

The morphological features were classified as lobulated shape in 8 cases (50.0%) of digit PNSTs (Figs 2, 3 and 5) and none (0.0%) of major-nerve PNSTs with significant difference (*P* = 0.001). Involvement of a digital neurovascular bundle was present in 13 (81.3%) cases of digit PNSTs (Fig 1) and absent in 3 (18.8%) digital PNSTs. The detailed MRI features in the two groups are described in Table 3.

**Table 3:** Detailed MRI findings of digital PNST.

	Digital PNST (n = 16)	Major-nerve PNST (n = 20)	*P*
T1WI			
mild hyperintense SI than muscle	9 (56.2%)	0 (0.0%)	< 0.001
isointense SI with muscle	7 (43.8%)	20 (100.0%)	
T2WI			
bright SI as fluid	1 (6.3%)	0 (0.0%)	0.444
hyperintense SI than muscle	15 (93.8%)	20 (100.0%)	
T2WI pattern			
heterogeneous	9 (56.3%)	20 (100.0%)	0.004
homogeneous	7 (43.8%)	0 (0.0%)	
Enhancement pattern			
heterogeneous	12 (75.0%)	20 (100.0%)	0.066
homogeneous	4 (25.0%)	0 (0.0%)	
Shape			
ovoid	8 (50.0%)	20 (100.0%)	0.001
lobular	8 (50.0%)	0 (0.0%)	
Digital Neurovascular involvement			
present	13 (81.3%)	N/P	N/P
absent	3 (18.8%)	N/P	

N/P, not performed; SI, signal intensity.

### Comparison of clinical and MRI findings between digital PNST subtypes

There was no significant difference in the presence of tenderness and Tinel sign between the digital PNST subtypes (*P* = 0.244 and 0.595, respectively).

None in the classic MRI findings of PNST showed the statistical differences between the digital PNST subtypes, but five cases of digital schwannoma showed fascicular sign (45.5%).

**Fig 6:**
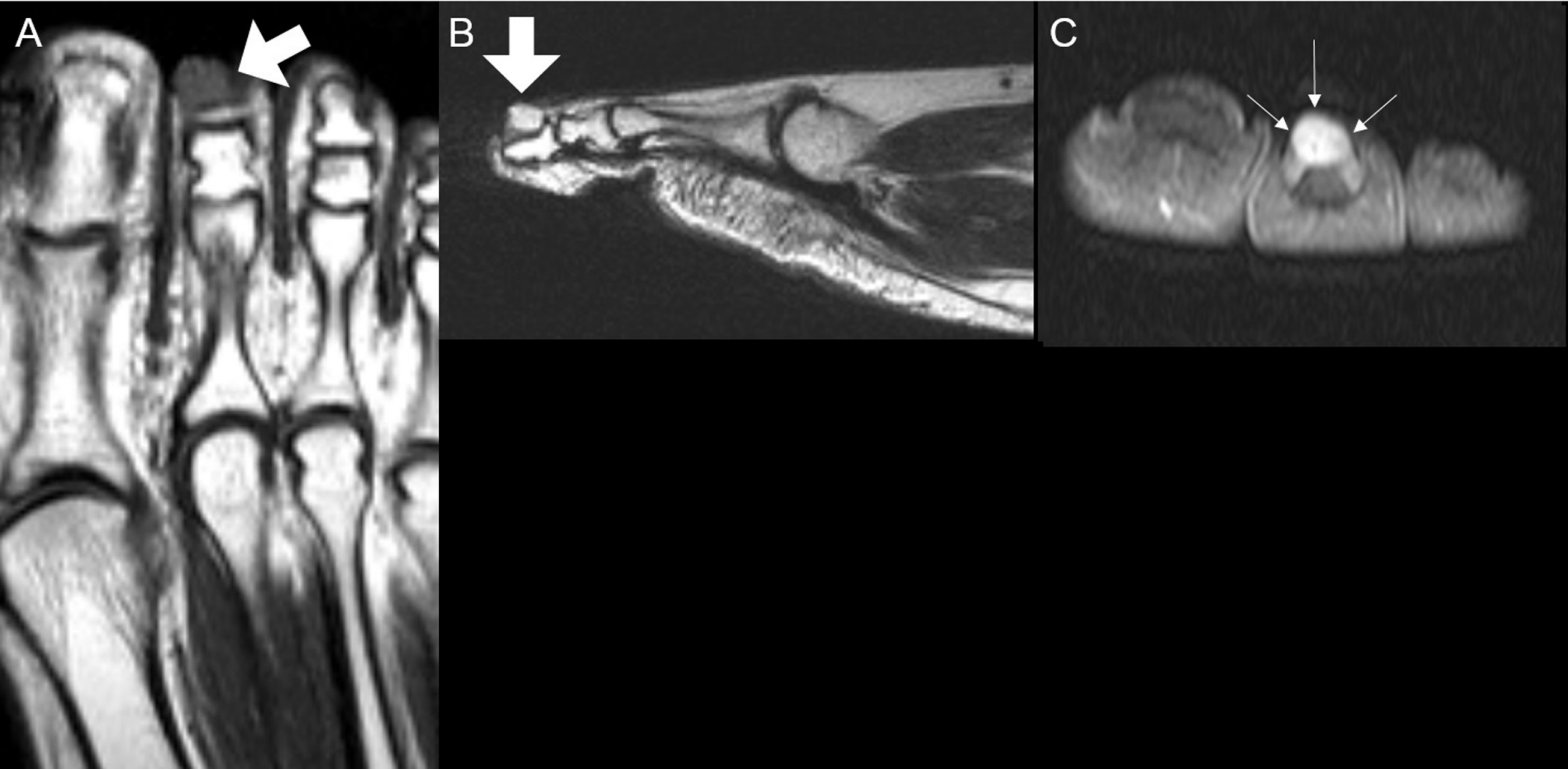
A 31-year-old woman with digital schwannoma in the subungual area of the 2^nd^ toe. (**A)** On coronal T1-weighted image, the mass demonstrates mild hyperintense SI compared to skeletal muscle (arrow). (**B)** On sagittal T2-weighted image, the mass reveals homogeneously hyperintense SI compared to skeletal muscle (arrow). (**C)** On axial contrast-enhanced T1-weighted image, the mass demonstrates homogeneous enhancement with displacement of the nail plate (thin arrows). ***SI***, signal intensity.

On precontrast T1-weighted images, mildly hyperintense SI compared to the skeletal muscle was noted in eight (72.7%) of digital schwannomas (Fig 6), one (25.0%) of digital neurofibroma (Fig 2), and none in digital ancient schwannoma without significant difference (*P* = 0.130). On T2-weighted images, bright SI as fluid was noted in one (25.0%) of digital neurofibroma (Fig 2), and none in digital schwannoma and ancient schwannoma without significant difference (*P* = 0.202). Although only three (27.3%) digital schwannomas (Figs 4 and 6) and none of digital ancient schwannoma showed homogeneity on T2-weighted images, all four (100.0%) digital neurofibromas (Fig 2) showed homogeneous SI on T2-weighted images with statistical difference (*P* = 0.028). On contrast-enhanced T1-weighted images, homogeneous enhancement was revealed in three cases (27.3%) of digital schwannoma (Figs 4 and 6), one (25.0%) digital neurofibroma, and none in digital ancient schwannoma without significant difference (*P* = 0.834).

The lobulated morphology was noted in four (36.4%) digital schwannomas (Fig 5), three (75.0%) digital neurofibromas (Fig 2), and one (100.0%) digital ancient schwannoma (Fig 3) without significant difference (*P* = 0.244). Involvement of digital neurovascular bundle was present in nine (81.8%) digital schwannomas (Fig 1), four (100.0%) digital neurofibromas, and none in digital ancient schwannoma without significant difference (*P* = 0.072). Table 4 demonstrates a summary of the clinical and MRI findings between the digital PNST subtypes.

**Table 4:** Comparison of clinical and MRI findings between digital PNST subtypes.

	Digital schwannoma (n = 11)	Digital neurofibroma (n = 4)	Digital ancient schwannoma (n = 1)	*P*
Tenderness	7 (63.6%)	1 (25.0%)	0 (0.0%)	0.244
Tinel sign	2 (18.2%)	0 (0.0%)	0 (0.0%)	0.595
Split fat sign	0 (0.0%)	0 (0.0%)	0 (0.0%)	N/A
Entering and exiting nerve	0 (0.0%)	0 (0.0%)	0 (0.0%)	N/A
Fascicular sign	5 (45.5%)	0 (0.0%)	0 (0.0%)	0.191
Target sign	0 (0.0%)	0 (0.0%)	0 (0.0%)	N/A
Thin hyperintense rim	0 (0.0%)	0 (0.0%)	0 (0.0%)	N/A
T1WI				0.130
mild hyperintense SI than muscle	8 (72.7%)	1 (25.0%)	0 (0.0%)	
isointense SI with muscle	3 (27.3%)	3 (75.0%)	1 (100.0%)	
T2WI				0.202
bright SI as fluid	0 (0.0%)	1 (25.0%)	0 (0.0%)	
hyperintense SI than muscle	11 (100.0%)	3 (75.0%)	1 (100.0%)	
T2WI pattern				0.028
heterogeneous	8 (72.7%)	0 (0.0%)	1 (100.0%)	
homogeneous	3 (27.3%)	4 (100.0%)	0 (0.0%)	
Enhancement pattern				0.834
heterogeneous	8 (72.7%)	3 (75.0%)	1 (100.0%)	
homogeneous	3 (27.3%)	1 (25.0%)	0 (0.0%)	
Shape				0.244
ovoid	7 (63.6%)	1 (25.0%)	0 (0.0%)	
lobular	4 (36.4%)	3 (75.0%)	1 (100.0%)	
Digital Neurovascular involvement				0.072
present	9 (81.8%)	4 (100.0%)	0 (0.0%)	
absent	2 (18.2%)	0 (0.0%)	1 (100.0%)	

N/A, not available.

### Unusual manifestations of digital PNSTs

Unusual manifestations, causing difficulty in the prediction of PNST, were found in three of the digital PNST cases but none in the major-nerve PNST. All of them occurred in the foot and were proven as two schwannomas and one ancient schwannoma. The unusual manifestations were bone destruction (Fig 3), skin thickening (Fig 5), and subungual location of the tumor (Fig 6).

## Discussion

The clinical symptoms and classic MRI findings of PNST were insufficient for the diagnosis of digital PNST. Among the cases of digital PNSTs, the only classic PNST MRI finding demonstrated was the fascicular sign, and only 31.3% of the cases manifested this sign. The frequency of clinical symptoms was also low (Tinel sign, 12.5%). Contrarily, the major-nerve PNST cases revealed more than two characteristic MRI findings of PNST, and most of the patients presented with Tinel sign (95.0%), combinations sufficient for the diagnosis of PNST. In our study, most of the digital PNSTs were soft tissue masses with slight hyperintense SI on T1- (56.2%), while none was noted in major-nerve PNST. Most of digital PNSTs demonstrated hyperintense SI on T2-weighted images (87.5%) with heterogeneous enhancement (75.0%), similar with those findings of major-nerve PNSTs. However, homogeneity was noted in 43.8% of digital PNSTs on T2-weighted image and homogeneous enhancement in 25% of digital PNSTs on contrast-enhanced T1-weighted image. Lobulated shape was noted in 50% of digital PNSTs, while none was noted in major-nerve PNSTs. Most digital PNSTs were located along the course of a digital neurovascular bundle (81.3%). Unusual manifestations, such as bone destruction, skin involvement, and subungual location, were found in the foot.

Digital PNSTs mostly occurs from digital nerves. The clinical and imaging findings of digital PNSTs have been reported as cases, but have not been described in a specific study [3, 5, 8]. Therefore, the clinical symptoms and MRI findings of them are still uncertain. Only two digital PNSTs (12.5%) presented neurologic sign in our study, so digital PNSTs are not easily recognized by physical examination. Furthermore, digital PNSTs were detected earlier than major-nerve PNSTs, even if they were smaller. We thought that digital PNSTs might be easily detected than major-nerve PNSTs due to superficial location.

Among the five classic MRI findings of PNST, digital PNST showed only the fascicular sign. Digital PNSTs did not reveal a split fat sign because they are located in the subcutaneous fat layer, which masks the fatty rind around tumor. Unlike major-nerve PNST, digital PNST did not the show entering and exiting nerve sign, and we thought that the digital PNST was too small to reveal this sign. In addition, the target sign and thin hyperintense rim were not revealed in digital PNST. We speculated that digital PNSTs are smaller in size than major-nerve PNSTs, so digital PNSTs might have relatively more uniform internal components than major-nerve PNSTs. Fascicular sign, a specific feature of schwannoma [15], was revealed in 31.3% of digital PNSTs, but this finding alone made it difficult to diagnose digital PNST. However, if the digital mass suggests a PNST, the fascicular sign within the mass favors the schwannoma more likely. In our study, the morphology, SI, and enhancement pattern of some digit PNSTs were different from those of major-nerve PSNTs in some ways. The morphology of digital PNSTs is relatively lobulated in half of cases. We thought that they showed lobulation because there is less anatomical space to grow in digit compared to the proximal portion of extremities. The differences in SI and enhancement pattern might be related with their histological composition such as compact hypercellular area (Antoni A) with relatively smaller hypocellular area (Antoni B) and lesser hyaline or cystic change. For these reasons, digital PNSTs might be difficult to diagnose based on MRI findings.

Various soft tissue masses which occur in the digit can be included in the differential diagnosis. Tenosynovial GCT is the most common soft tissue tumor of the digit. Tenosynovial GCT shows a characteristic “blooming” of hemosiderin on gradient-echo imaging and is more related to the tendon with an obtuse angle [20]. On the contrary, the digital PNSTs in the present study showed different SI and occasionally abutted the tendon sheath with an acute angle. Angioleiomyoma and hemangioma are other tumors that can be included in the differential diagnosis. Angioleiomyoma is a well-demarcated subcutaneous mass of isointense or slight hyperintense SI on T1-weighted images and heterogeneously hyperintense SI on T2-weighted images with homogeneously strong enhancement and characterized with an adjacent tortuous vascular structure [21, 22]. Soft tissue hemangiomas show variable SIs according to the internal compositions, such as fat, fibrous, myxoid, smooth muscle, thrombus, or phlebolith [23]. However, when a hemangioma involves the digit, it might show a small soft tissue mass with bright SI on T2-weighted images, as that of vessel, and strong enhancement in contrast-enhanced T1-weighted images [24]. On T2-weighted images, digital PNST showed hyperintense SI, but less than a vessel, and on contrast-enhanced T1-weighted images, it showed heterogeneous enhancement. However, 43.8% of digital PNSTs showed homogeneity on T2-weighted images and 25% of digital PNSTs showed homogeneous enhancement. Even in these cases, digital PNST show neither connected vessels nor surrounding vascular engorgement. These findings might be helpful for the differentiation of digital PNST from angioleiomyoma or hemangioma. Although digital neurovascular involvement might be a non-specific finding because many soft tissue masses of the digit are usually related to the neurovascular bundle due to the small space in the digit, if the axis of the digital tumor is carefully considered, it might be useful to differentiate digital PNST from other soft tissue tumors. It can be assumed that the axis of a digital PNST is more parallel and closer to the axis of the neurovascular bundle than the axis of other digital tumors. This can be inferred from the difference in whether or not the digit tumors are directly related to the digital nerve.

Three of 9 digital PNSTs cases in the foot (33.3%) were associated with unusual manifestations, including bone destruction, skin thickening, and subungual location, and all cases were schwannoma. In previous studies, all reported cases of bone invasion from schwannoma involved the foot [25, 26]. Compared to a mass on the hand, a mass on the foot may be difficult to recognize until it grows enough to destroy the bone. Schwannomas can involve bone by three mechanisms [27]. First, the tumor may occur at the bone centrally. Second, the tumor may arise in the nutrient canal of bone, enlarging the canal. Finally, an extraosseous soft tissue may cause secondary bony erosion. The aggressiveness of the tumor can raise suspicion for malignancy, such as a soft tissue sarcoma. However, slow-growing schwannomas typically have a well-defined margin with a thin low-signal rim, suggesting epineurium. Despite bone destruction, normal fatty bone marrow is preserved, suggesting secondary pressure erosion by benign large soft tissue tumors. Skin thickening in schwannoma is extremely rare, and it is difficult to differentiate between skin involvement of tumor and reactive skin thickening on the MRI. Cutaneous schwannomas are typically asymptomatic and may originate from a terminal cutaneous nerve [28, 29]. Dermatofibrosarcoma protuberans was considered in the differential diagnosis for skin thickening. Dermatofibrosarcoma protuberans in an acral site is rare, but a few cases were reported at the toe [30, 31]. Although it is difficult to differentiate between cutaneous schwannoma and dermatofibrosarcoma, cutaneous schwannoma has a low SI rim, suggesting epineurium, which is not seen in dermatofibrosarcoma. Subungual schwannoma is also extremely rare [32]. A few cases of subungual schwannomas in the toe and finger have been reported in the literature, and the reports only described the sonographic findings [32–34]. The MRI findings in our cases were similar to those of a glomus tumor, but the absence of symptoms unique to glomus tumors, such as severe pain, intense tenderness that may be provoked by mild trauma, and temperature sensitivity, was helpful in making the diagnosis. Our case presented with pain, but it was mild. Because of the differences in the degree of pain, the size of the tumor at the time of detection might be different between subungual glomus tumor and PNST. The former might be found when small due to severe pain, while the latter might be detected when relatively large due to mild pain [32]. The nail change may be combined in subungual schwannoma such as lift, thinning, and tearing [32, 33].

The limitations of our study include its small sample size and retrospective nature. Furthermore, although we have described as many differential diagnoses as possible, it might be difficult to suspect digital PNST in clinical practice if it shows unusual findings. Despite these limitations, our study encourages an expansion of the spectrum of imaging findings for digital PNST.

In conclusion, Tinel sign is not common in digital PNSTs. The classic findings of PNST are less commonly observed in digital PNST, with the exception of the fascicular sign. Digital PNSTs frequently show different shape, SI, and enhancement pattern from major-nerve PSNTs on MRI. However, the digital soft tissue tumors involving digital neurovascular bundle and especially presenting a fascicular sign, digital PNSTs should be considered in the differential diagnosis.

## Supporting information

S1 File (XLSX)

S1 Table Logistic regression analysis between two groups against MRI findings. (DOCX)

S2 Table Comparison of MRI findings between digital schwannoma and major-nerve schwannoma. (DOCX)
