# Supplementary material for: Benign peripheral nerve sheath tumor of digit versus major-nerve: Comparison of MRI findings
Source: PLoS One. 2020 Mar 26;15(3):e0230816. doi: 10.1371/journal.pone.0230816 (PMC7098591; doi:10.1371/journal.pone.0230816)
Supplement: S1 Table — (DOCX) [file pone.0230816.s002.docx]

Logistic regression analysis between two groups against MRI findings

|  | **Digit PNST**  **(n = 16)** | **Major-nerve PNST**  **(n = 20)** | **Odds ratio** | | **Age-adjusted Odds ratio** |
| --- | --- | --- | --- | --- | --- |
| Split fat sign | 0 (0.0%) | 20 (100.0%) | N/A | | N/A |
| Entering and exiting nerve | 0 (0.0%) | 19 (95.0%) | N/A | | N/A |
| Fascicular sign | 5 (31.3%) | 18 (90.0%) | 0.024 (0.002-0.248) | | 0.024 (0.002-0.248) |
| Target sign | 0 (0.0%) | 2 (10.0%) | N/A | | N/A |
| Thin hyperintense rim | 0 (0.0%) | 9 (45.0%) | N/A | | N/A |
| N/A: not available | | | |  | |
