# Supplementary material for: Benign peripheral nerve sheath tumor of digit versus major-nerve: Comparison of MRI findings
Source: PLoS One. 2020 Mar 26;15(3):e0230816. doi: 10.1371/journal.pone.0230816 (PMC7098591; doi:10.1371/journal.pone.0230816)
Supplement: S2 Table — (DOCX) [file pone.0230816.s003.docx]

Comparison of MRI findings between digital schwannoma and major-nerve schwannoma

|  | **Digital schwannoma**  **(n = 11)** | **Major-nerve schwannoma (n = 19)** | **P** |
| --- | --- | --- | --- |
| Split fat sign | 0 (0.0%) | 19 (100.0%) | <0.001 |
| Entering and exiting nerve | 0 (0.0%) | 19 (100.0%) | <0.001 |
| Fascicular sign | 5 (45.5%) | 18 (94.7%) | 0.002 |
| Target sign | 0 (0.0%) | 2 (6.5%) | 0.510 |
| Thin hyperintense rim | 0 (0.0%) | 11 (57.9%) | 0.001 |
| Shape |  |  | 0.005 |
| ovoid | 7 (63.6%) | 19 (100.0%) |  |
| lobular | 4 (36.4%) | 0 (0.0%) |  |
